# Neutralizing Antibodies Against a Specific Human Immunodeficiency Virus gp41 Epitope are Associated With Long-term Non-progressor Status

**DOI:** 10.1016/j.ebiom.2017.07.007

**Published:** 2017-07-11

**Authors:** Olivier Lucar, Bin Su, Valérie Potard, Assia Samri, Brigitte Autran, Christiane Moog, Patrice Debré, Vincent Vieillard

**Affiliations:** a Sorbonne Universités, UPMC Univ Paris 06, INSERM U1135, CNRS ERL8255, Centre d'Immunologie et des Maladies Infectieuses (CIMI-Paris), Paris, France; b U1109 INSERM, FMTS, Université de Strasbourg, Strasbourg, France; c Center for Infectious Diseases, Beijing You'an Hospital, Capital Medical University, Beijing 100069, China; d Sorbonne Universités, UPMC Univ Paris 06, UMR-S 1136, Paris, France

**Keywords:** HIV-1, Long-term non-progressors, Neutralizing antibody

## Abstract

Antibodies (Abs) play a central role in human immunodeficiency virus (HIV) protection due to their multiple functional inhibitory activities. W614A-3S Abs recognize a specific form of a highly conserved motif of the gp41 envelope protein and can elicit viral neutralization to protect CD4^+^ T cells. Here, we describe in detail the neutralizing profile of W614A-3S Abs in untreated long-term non-progressor (LTNP) HIV-infected patients. W614A-3S Abs were detected in 23.5% (16/68) of untreated LTNP patients compared with < 5% (5/104) of HIV-1 progressor patients. The W614A-3S Abs had efficient neutralizing activity that inhibited transmitted founder primary viruses and exhibited Fc-mediated inhibitory functions at low concentrations in primary monocyte-derived macrophages. The neutralizing capacity of W614A-3S Abs was inversely correlated with viral load (*r* = − 0.9013; *p* < 0.0001), viral DNA (*r* = − 0.7696; *p* = 0.0005) and was associated the preservation of high CD4^+^ T-cell counts and T-cell responses. This study demonstrates that W614A-3S neutralizing Abs may confer a crucial advantage to LTNP patients. These results provide insights for both pathophysiological research and the development of vaccine strategies.

## Highlights

• Long-term non-progressor patients produce W614A-3S neutralizing antibodies (NAb).
• Neutralizing capacity of W614A-3S was correlated with viral load, CD4 count, and T-cell responses.
• W614A-3S NAbs can be used in functional “cure” and vaccine strategies.

Long-term non-progressors (LTNPs) are individuals infected with HIV, who maintain a high CD4 count without antiretroviral therapy. Understanding the mechanisms implicated in this process will help in developing efficient HIV vaccine. We show that in contrast to treated HIV-1-infected patients, LTNPs individuals produce specific antibodies able to control the virus while preserving CD4 count and T-cell responses. This could form the basis for the development of future treatments or vaccine strategies based on W614A-3S to fight HIV-1.

## 1 Introduction

Neutralizing antibodies (NAbs) are a good correlate of protection in infectious diseases such as yellow fever, smallpox, and measles (Amanna et al., 2008). The potential protective role of Nabs during the course of human immunodeficiency virus (HIV) infection remains a highly debated issue. Many HIV-infected individuals naturally develop NAbs that target several sites on the gp41 and gp120 HIV-1 envelope proteins after several years of infection, but only 10–25% develop potent and broadly reactive Nabs (Mikell et al., 2011, Hraber et al., 2014, West et al., 2014). These findings suggest that the human immune system can achieve NAb responses, but whether these Abs are naturally protective during HIV infection remains unclear.

Studies of NAbs in general have provided an enormous impetus to HIV vaccine research and to immunology as a whole (Sadanand et al., 2016). Monoclonal Abs (mAbs) with the remarkable ability to neutralize most circulating strains of HIV-1 were recently isolated from HIV-infected individuals. Examples include 3BNC117 and PGT121 mAbs, which both transiently block infection and suppress viremia in simian/HIV (SHIV)-infected macaques, demonstrating that the passive administration of potent NAbs protects macaques for a short period of time (Barouch et al., 2013). In HIV-1-infected humans, a single infusion of 3BNC117 mAb, which specifically targets the CD4-binding site on gp120, decreased viremia for up to 28 days (Caskey et al., 2015).

Although these data indicate the potential effectiveness of passive immunotherapy by NAbs, the successful development of preventive HIV-1 vaccines requires a thorough understanding of how Nabs are induced during HIV-1 infection. The unusual maturation features of NAbs make them extremely difficult to induce and indicate that one of the most important challenges for vaccines development is to characterize well-defined targets that specifically stimulate neutralizing activity (Sadanand et al., 2016). The gp41 subunit is far more conserved than gp120, and the fusion machinery is common to all strains. We previously described a highly specific motif localized in a gp41 HIV-1 region, called 3S (Vieillard et al., 2005, Vieillard et al., 2016), localized between the N-terminal heptad repeat (HR) 1 and the HR2, that appears to be exposed to the surface in the trimeric pre-fusion structure of the HIV-1 envelope (Fig. S1), in line with recent resolve structures of the HIV-1 envelope trimer (Pancera et al., 2014, Lee et al., 2016). In an *in vivo* macaque model, immunization with a candidate vaccine based on the 3S motif induced non-neutralizing Abs, which limited CD4^+^ T-cell depletion, immune activation, and inflammation, thus achieving immune protection and restoring immune homeostasis (Vieillard et al., 2008, Vieillard et al., 2012).

An alanine-scanning assay within the 3S motif of the viral gp41 protein showed that a tryptophan residue at position 614 (W614) is crucial for the virus entry (Petitdemange et al., 2013). The main reason could be that this region plays a key role in the formation of the six-helix bundled gp41 ectodomain core structure that imposes several kinetic and steric constraints responsible for the high degree of motif preservation, as previously reported (Gallo et al., 2003). Altogether, these data could explain the absence of detectable “3S” escape variants, and the remarkable conservation of the 3S motif within the gp41 (Vieillard et al., 2005, Potard et al., 2013). Next, we generated in mice a class of Abs against the 3S motif, called W614A-3S Ab that elicits neutralizing activity, against a panel of tier 1 and tier 2 viruses from clades A, B, C and E (Petitdemange et al., 2013). More recently, these data were confirmed in rabbit and macaque models (unpublished data are from Vieillard et al.). Our data are in accordance with Bradley et al. (2016) showing that amino-acid changes in gp41 membrane proximal region (MPER) induce viral neutralization sensitivity. Interestingly, we also determined that approximately 5% of HIV-1-infected progressor patients naturally produce neutralizing W614A-3S Abs (Petitdemange et al., 2013).

In this study, we analyzed the neutralizing activity of W614A-3S Abs isolated from long-term non-progressor (LTNP) patients from the French ALT (“Asymptomatic Long-Term”) cohort (“Agence Nationale de Recherche sur le Sida” ANRS CO15). These HIV-1-infected individuals account for < 0.4% of the total HIV population (Grabar et al., 1999). They maintain high CD4^+^ T-cell counts and remain therapy naïve. Here, we report an unusually high frequency of LTNP patients with W614A-3S Nabs. These Abs displayed neutralizing activities over a five-year follow-up period, which are correlated with low viral load, low viral reservoir, and high CD4^+^ T-cell responses. These data support the hypothesis that these specific Nabs play an important role *in vivo* in maintaining LTNP status.

## 2 Materials and Methods

### 2.1 Study Subjects

This study enrolled 68 LTNP HIV-1^+^ patients from the French ALT cohort (ANRS CO15). Their characteristics are summarized in Table 1. As previously reported, ALT cohort members met the following inclusion criteria: HIV seropositivity for at least 8 years and CD4 cell counts > 600/mm^3^ for the past 5 years, whatever the viral load, but without symptoms or antiretroviral therapy (Grabar et al., 1999). LTNP patients were subdivided into three groups according to their capacity to produce neutralizing W614A-3S Abs, non-neutralizing WT-3S^+^ Ab only, or neither subtype (Neg). The CO15 ALT cohort is funded and sponsored by ANRS and was approved by the ethics review committee of Ile de France – VI. All patients provided written informed consent, and all methods were performed in accordance with relevant guidelines and regulations indicated by the Declaration of Helsinki.

**Table 1:** Characteristics of study ALT patients.

Characteristics	Neg	WT-3S	W614-3S	P⁎
Number	24	28	16	
Median age in years	36.0	36.6	38.0	0.55
Male:Female (n)	20:4	24:4	8:8	**0.02**
Median viral load (quartiles)	9.3 × 10^3^ (2.1 × 10^3^–1.0 × 10^5^)	2.3 × 10^5^ (4.5 × 10^3^–1.0 × 10^5^)	1.3 × 10^2^ (3.3 × 10^1^–4.1 × 10^2^)	**< 0.0001**
Median viral DNA (quartiles)	403 (120–1449)	430 (114–1011)	24 (8–27)	**< 0.0001**
Median CD4 count (quartiles)	605 (467–769)	717 (609–799)	697 (590–833)	0.09
Median % CD4 (quartiles)	33.0 (27.5–37.0)	30.0 (26.0–36.0)	39.5 (35.0–42.5)	**0.01**

In CD4^+^ T cells (%, median, quartiles)				
HLA-DR^+^	8.5 (4.0–9.0)	8.5 (6.0–14.0)	6.0 (3.0–11.0)	0.12
CD38^+^	76.5 (70.0–86.0)	76.0 (58.0–80.5)	69.5 (50.0–75.0)	0.07
CD45Ra^+^	59.0 (49.0–65.0)	53.0 (43.5–91.0)	51.0 (41.0–68.0)	0.24
CD45Ro^+^	47.0 (35.0–58.5)	50.0 (35.5–76.0)	51.0 (39.0–66.0)	0.81
Median CD8 count (quartiles)	818 (685–964)	1121 (974–1541)	717 (531–1168)	**0.004**
Median % CD8 (quartiles)	42.5 (38.0–49.5)	48.0 (41.5–60.0)	39.0 (36.0–44.0)	**0.004**

In CD8^+^ T cells (%, median, quartiles)				
HLA-DR^+^	27.0 (19.0–35.0)	33.0 (28.0–45.0)	22.0 (16.0–30.0)	**0.007**
CD38^+^	56.5 (45.0–69.0)	51.0 (38.0–66.0)	43.5 (37.0–50.0)	**0.03**
CD45Ra^+^	65.0 (54.0–74.5)	54.0 (46.0–70.0)	64.0 (55.0–74.0)	0.24
CD45Ro^+^	28.5 (20.2–41.2)	35.0 (24.2–45.7)	30.0 (21.0–38.0)	0.52
CD57^+^	38.0 (34.0–52.0)	34.0 (26.5–83.0)	34.0 (20.0–43.0)	0.49

Significant values (P < 0.05) expressed in *bold*.

⁎ Statistical analysis was performed using chi-square test for categorical variables and Kruskal-Wallis test for continuous variables, with Dunn post-test.

### 2.2 CD4 Count and Viral Production

The course of the immune and virological status of LTNP patients from the ALT cohort has been described elsewhere (Candotti et al., 1999, Magierowska et al., 1999, Martinez et al., 2005). All tests were conducted in a single laboratory. CD4 cell counts were performed on fresh blood by flow cytometry (Coulter) and were determined in accordance with a standard internal control (mean ± SD reference value, 858 ± 260 cells/mm^3^). HIV-1 RNA viral load was quantified in fresh plasma samples using an ultrasensitive HIV-1 Amplicor-Monitor assay (Roche-Diagnostic Systems; limit of detection, 20 copies/ml). The level of HIV-1 viral DNA was determined in frozen peripheral blood mononuclear cells (PBMCs) using a modified Amplicor Monitor assay (Roche Laboratories) with an internal HIV-1 proviral DNA standard. Results were expressed as copies of HIV-1 viral DNA/10^6^ PBMCs with a limit of detection of 1 copy/10^6^ PBMCs.

Sequencing analysis of the patient viral sequences at the 614 position of the 3S motif is shown in Table S1. This analysis came from the complete protein sequence of gp41 from the 56 available samples of LTNP patients from the ALT cohort (Potard et al., 2013).

### 2.3 Phenotyping and Specific CD4^+^/CD8^+^ T-cell Responses

CD4^+^ and CD8^+^ T cells were analyzed on a Beckman Coulter FC500 flow cytometer using the CXP Analysis (Beckman Coulter) software, with an appropriate combination of antibodies: CD4-PE-Cy7 (#SK3), CD8-PE-Cy7 (#RPA-T8), HLA-DR-PE (#G46–6), CD38-PE (#HIT-2), CD57-APC (#NK-1) from Becton Dickinson, CD45-RA-ECD (#2H4), and CD45-RO-FITC (#UCHL1) from Beckman Coulter.

CD8^+^ T-cell responses were evaluated using a standard IFN-γ enzyme-linked immunospot assay. PBMCs in triplicate were incubated with medium alone (negative control), phytohemagglutinin (positive control), or pools of 15-mer peptides overlapping by 11 amino-acids and spanning the entire Gag, RT and Nef HIV-1 proteins (2 μg/ml), previously described (Xie et al., 2010). Results are expressed as spot-forming cells (SFC)/10^6^ PBMCs. The positive threshold was 50 SFCs/10^6^ PBMC after background subtraction.

For the CD4^+^ T-cell proliferation assay, PBMCs were stimulated with media, HIV-p24 protein (0.25 μg/ml; donated by Transgene, Strasbourg, France), or recall antigens, specifically varidase (5 μg/ml; Sigma-Aldrich) or tuberculin (purified protein derivative, PPD) (1 μg/ml; Statens Serum Institut, Denmark). After a 6-day incubation, 1 γCi of [^3^H]-thymidine was added to each well, and the plate was incubated for 18 h. The stimulation index (SI) was calculated as the ratio of (cells plus stimuli) cpm to (cells plus medium) cpm. Positive antigen-specific responses were defined as those with > 3000 cpm and an SI > 3.

### 2.4 ELISA, Affinity Purification of Antigen-specific IgG, and Viral Neutralization

Plasma samples were titrated by ELISA with EIA/RIA 96-well flat-bottom plates coated overnight with 100 ng/well of purified 3S peptides NH2-pwnasSWSNKSssleqiw-COOH or NH2-pwnasSASNKSssleqiw-COOH to specifically quantify WT-3S and W614A-3S Abs, respectively, as described (Vieillard et al., 2006, Petitdemange et al., 2013). A scramble peptide NH2-pnsakwlwssiqsnswes-COOH served as control (Fig. S2). Purified peptides were prepared by Covalab (Villeurbanne, France). Specific Abs were purified from heat-inactivated plasma from HIV-infected patients, as described (Cheung et al., 2012, Petitdemange et al., 2013). Briefly, total IgG from the serum was purified using Protein A sepharose beads (GE Healthcare), then incubated rotating for 15 min in a column with the immunogen peptide covalently coupled to sepharose beads. By gravity flow, the unbound fraction was drained, and the column was washed extensively with 1 x PBS to eliminate nonspecific IgG. Antigen-specific polyclonal IgG pool was eluted sequentially with 0.1 M glycine/HCl buffer at pH 3.5, followed by pH 2.7, and finally pH 1.8. Each elution was immediately neutralized with 1 M Tris buffer (pH 8.5). Purified Abs were dialyzed against PBS (Slide-A-Lyzer Dialysis cassette, Pierce) and then quantified by NanoDrop using a BCA (BiCinchoninic acid Assay) assay with bovine gamma globulin as the reference standard (Thermo Fisher). The specificity of WT-3S and W614A-3S Ab was tested by Elisa after each set of purification with specific and control synthetic peptides (scramble, WT-3S and W614A-3S peptides). Negatives values were < 0.15 OD_450_ and positives values ≥ 1.0 OD_450_.

Neutralization by purified W614A-3S Abs was assessed at starting concentrations of 2 μg/ml, followed by five 2-fold dilutions with a standard TZM-bl assay against various tier 1 (NDK, NL4.3, SF162) and tier 2 (JR-CSF, YU2, QH0692) HIV-1 clade B strains, as well as two T/F molecular clones (pCH058 and pCH077), as described (Petitdemange et al., 2013, Heyndrickx et al., 2012). TZM-bl cells expressing Fc-gamma receptor 1 (FcγRI) (TZM-bl-Fc/FcγRI) were used to measure the FcγRI function of purified W614A-3S Abs, in the presence of SF162 and QH0692 HIV-1 strains (Perez et al., 2013). The panel of mAbs shown in Table S2 was used as controls. The following reagents were obtained from the NIH AIDS Reagent Program, Division of AIDS (NIAID, Germantown, MD, USA): TZM-bl (Cat # 8129) and TZM-bl-Fc/FcγRI (Cat # 11798) cells, as well as VRC01, VRC07, 3BNC117, PGT 121,447-52D, IgG1 b12 and 10-E8 mAbs. The 2F5 and 2G12 mAbs were obtained from Polymun Scientific (Austria), and the 10-1074 mAb was a gift from Dr. H. Mouquet (Pasteur Institute, Paris, France).

For the MDM assay, magnetic separation with the CD14 MicroBeads kit (Miltenyi Biotec) was used to purify monocytes from peripheral mononuclear cells from HIV-seronegative healthy donors. After the monocyte purity was determined by flow cytometry, the monocytes were cultured in AIM-V FCS-free medium with Glutamax 1 × (Thermo Fisher) and 100 U/ml GM-CSF (PeproTech). The culture medium was changed after 3 days of culture. On day 5, macrophages were washed twice before infection with BaL (subtype B, HIV-1 R5 strain), detailed elsewhere (Holl et al., 2004, Lederle et al., 2014). Various HIV-specific mAbs were used as controls in both the TZM-bl and MDM neutralizing assays, previously described (Holl et al., 2006).

### 2.5 Sample Preparation, Mass Spectometry Analysis of CDR-H3 Motifs

F(ab)2 fragments were prepared from affinity-purified W614A-3S Nabs and WT-3S non-neutralizing Abs using immobilized pepsin (Pierce, IL, USA). The pepsin-agarose beads were separated by applying the reaction solution to an Ultrafree centrifugal filter column (Millipore, MA, USA). F(ab)2 were applied to Econo-Pac chromatography column in gravity mode (BioRad). The elution fractions were trypsin digested at 37 °C for 60 min in a reaction solution (50% v/v TFE, 50 mM ammonium bicarbonate and 2.5 mM DTT), as described (Cheung et al., 2012, Wine et al., 2013). Trypsin digestion generates peptide fragments with enough coverage of the CDR-H3 region and of lengths appropriate for MS detection (Wine et al., 2013). Digested peptides were desalted by STAGE (Stop and go extraction) TIPS, and analyzed by LC-MS (Liquid chromatography-mass spectrometry)/MS.

LC-MS/MS was done by Alphalyse (Odense, Denmark), using the LTQ Orbitrap Velos (Thermo Fisher) mass spectrometer. The samples were loaded for 10 min using a Famos autosampler (LC Packings) onto a hand-poured fused silica capillary column (125 mm internal diameter × 20 cm) packed with Magic C18aQ resin (5 μm, 200 Å) using an Agilent 1100 series binary pump with an in-line flow splitter. The resulting MS/MS spectra were searched against a protein sequence database consisting of the in-house human VH sequences, and identified using the SEQUEST algorithm (Proteome Discoverer, Thermo Scientific). For post-acquisition analysis, passing peptides were remapped to the NGS (next-generation sequencing) immunoglobulin database, and matches were limited to those arising from expected cleavage site for trypsin. We therefore considered all Iso/Leu sequence variants as a single group, and mapped the group to all CDR-H3s associated with any of the group members, as described (Wine et al., 2013). For other isobaric pairings, only the top-ranked PSM (peptide spectrum matches) determined by the SEQUEST-Percolator pipeline were considered. Resulting sequences of CDR-H3 were then aligned with the following panel of monoclonal NAbs, used as controls: 2F5, 10-1074, 10E8, PGT121 (Tables S2 and S3).

### 2.6 Statistical Analysis

Characteristics of all patients of the ALT cohort were analyzed for the following 3 groups of patients: negative, WT-3S^+^, and W614A-3S^+^. Categorical variables were described as numbers and percentages and continuous variables as medians and interquartile ranges. All data accruing were taken into account. Categorical variables were tested with the χ2 test and continuous variables with the Kruskal-Wallis test.

Kaplan-Meier curves and log-rank tests were used to compare the probability of maintaining non-treated status in the 3 groups of patients described above. An univariate Cox regression model was used to assess the influence of W614A-3S Abs on the second endpoint. Time was right-censored at 5 years. Statistical significance was defined as a *p*-value < 0.05. SAS software (v9.3; SAS Institute Inc., Cary, NC, USA) was used for these statistical analyses.

## 3 Results

### 3.1 High Frequency of LTNP Patients with W614A-3S Neutralizing Abs

**Fig. 1:**
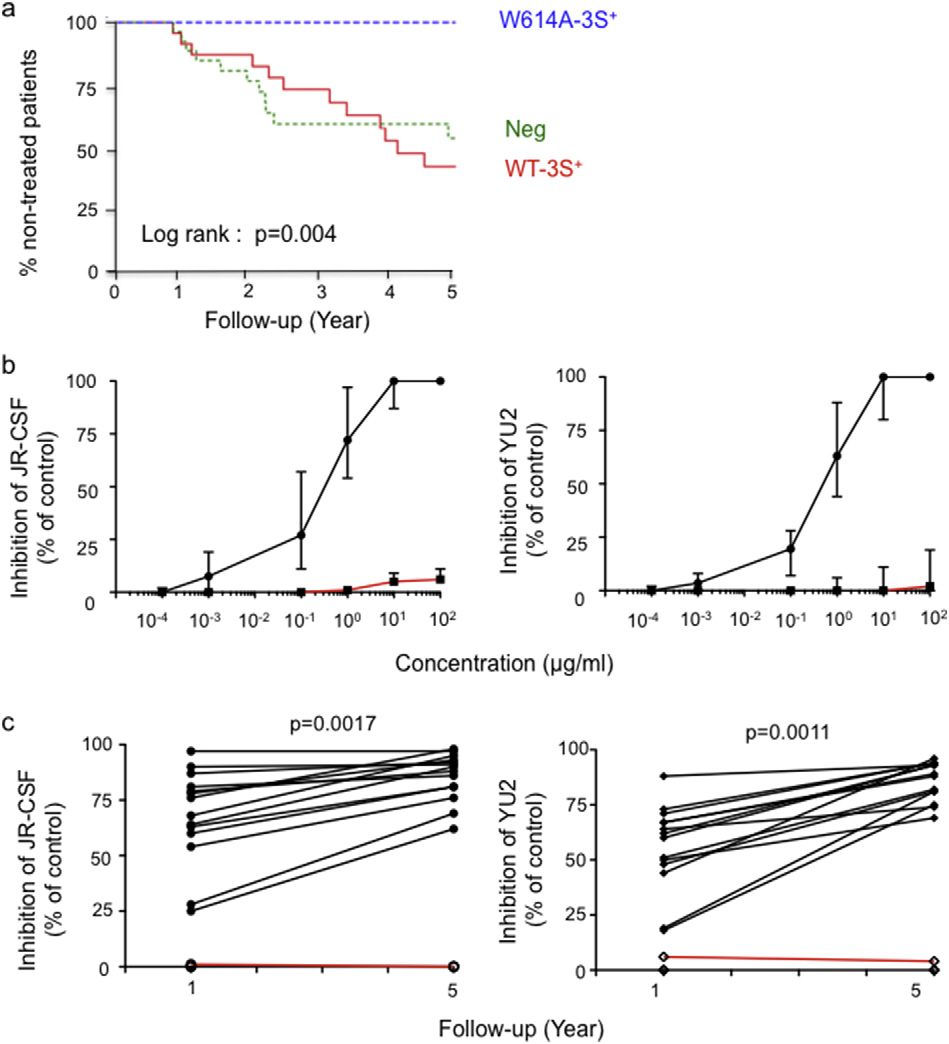
High frequency and neutralizing activity of W614A-3S Ab in LTNP patients. (A) Kaplan-Meier curves of the frequency of patients in the W614A-3S^+^ group (*n* = 16) compared with the WT-3S^+^ (*n* = 28) and Neg control (*n* = 24) groups. Blue: W614A-3S^+^ group; green: WT-3S^+^ group, and red: negative control group. (B) Dose-response curves of neutralizing activity at year 1 after cohort entry. Inhibition of infection by JR-CSF and YU2 HIV-1 strains, at different concentrations with all purified W614A-3S Abs (*n* = 16; closed symbols, black curves) or WT-3S Abs (open symbols, red curves, purified from 5 WT-3S patients), as control. Neutralizing activities were performed in the TZM-bl assay with concentrations of purified Abs from 0.00001 to 100 μg/ml. Data expressed in median ± range. These data are representative of 3 independent experiments. C, Evolution of the viral neutralization efficiency of purified W614A-3S Abs from the 16 ALT patients against JR-CSF and YU-2 HIV-1 strains at years 1 and 5 after cohort entry. Data are expressed as the percentage of decreased RLU values at 1 μg/ml W614A-3S Abs, compared with the RLU detected in infected cells without Abs (control). Similar data were obtained with other HIV-1 strains (NL4.3, and NDK) (data not shown). The red curve corresponds to the results obtained in the presence of the same amount of purified non-neutralizing WT-3S Abs. (For interpretation of the references to color in this figure legend, the reader is referred to the web version of this article.)

To study the role of W614A-3S Abs in the 68 LTNP patients, samples were subdivided into three groups according to their capacity to produce neutralizing W614A-3S Abs (*n* = 16; 23.5%), non-neutralizing WT-3S Abs only (*n* = 28), or neither 3S subtype (*N* = 24) at the time of enrollment in the cohort (Table 1 & Fig. S2). Notably, we observed that anti-WT-3S were IgG2 > IgG1 > IgG3, whereas anti-W614A-3S responses were IgG1 > IgG3 > IgG2, with IgG1 > 85%. The concentration of IgG4 was below detection level (data not shown). This switch of Ig subtype supports a possible maturation of the Ab response against the 3S epitope. Consistently, almost all broadly NAbs identified to date have either been isolated or expressed as IgG1 (Sadanand et al., 2016). Kaplan-Meier survival curves indicated that all W614A-3S^+^ patients remained non-treated at 5 years compared with 41.7% [20.5%; 61.7%] in the Neg group, and 53.1% [30.8%; 71.2%] in the WT-3S group (log rank: *p* = 0.004; Fig. 1A).

After immunoprecipitation to purify the W614A-3S Abs, dose-response curves of neutralizing activity were assessed at year 1 after cohort entry. Fig. 1B shows neutralizing activity against JR-CSF and YU-2 HIV-1 strains from all patients that produced W614A-3S Abs, whereas WT-3S Abs were not neutralizing, as previously observed (Vieillard et al., 2006, Petitdemange et al., 2013). More importantly, the potency of W614A-3S NAbs increased significantly 5 years later against the HIV-1 strains JR-CSF (*p* = 0.0017) and YU2 (*p* = 0.0011) (Fig. 1C). W614A-3S Abs exhibited high neutralizing activity against a panel of tier 1 (SF162), tier 2 virus and pseudo-viruses (JR-CSF, YU-2, QH092), two T/F infectious molecular clones (pCH058 and pCH77), but also BaL strain (Table 2). The IC_80_ levels, ranged from < 0.1 to 3.0 μg/ml, depending on the HIV-1 strain (Table 2) were similar or superior to those observed with a reference panel of potent neutralizing mAbs (Table S2).

**Table 2:**
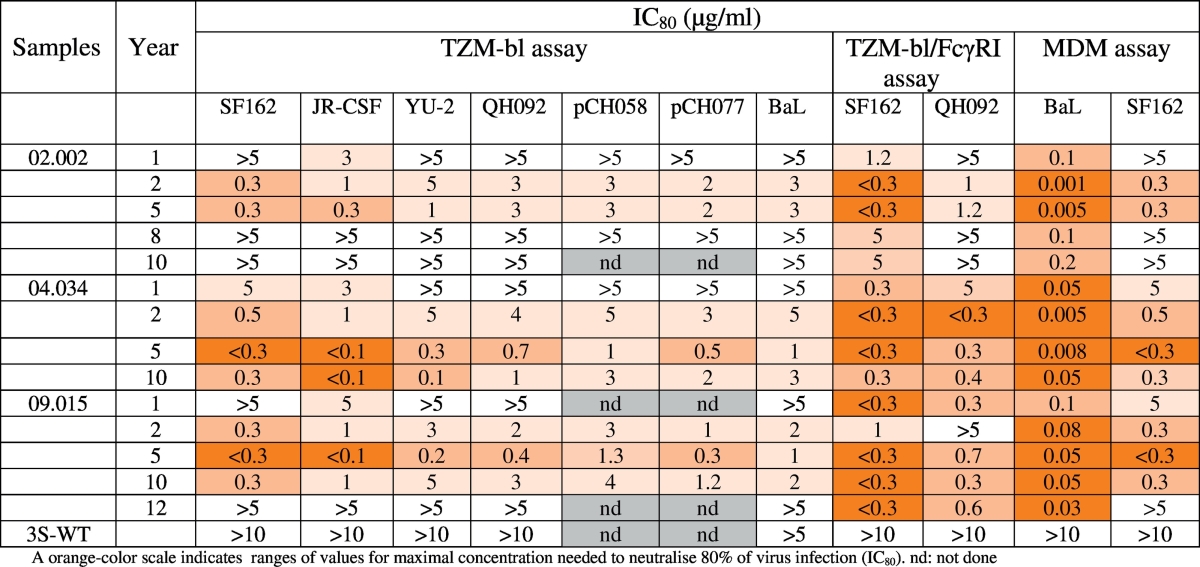
Neutralizing activity of purified W614A-3S Abs from ALT patients.

Next, we assessed the neutralizing capacity of W614A-3S Abs in the presence of TZM-bl/FcγRI cells, and observed that it was approximately twice that against standard TZM-bl targets (Table 2), suggesting an Fc-mediated inhibitory activity against tier 1 (SF162) and tier 2 (QH092) HIV-1 viruses. A similar differential response was previously reported for two other anti-gp41 NAbs, 2F5 and 4E10, which recognize distinct epitopes within the membrane-proximal external region (MPER) (Perez et al., 2013). The capacity of W614A-3S Abs to inhibit the infection of monocyte-derived macrophages (MDMs) was next assessed to detect both neutralization and Fc-mediated functions (Holl et al., 2004). The different purified W614A-3S Abs produced very strong inhibitory titers against MDMs infected by the HIV-1-BaL and SF162 strains (Table 2), similar or higher to those observed with a reference panel of potent neutralizing mAbs (Table S2). Taken together, these data suggest that W614A-3S Abs mediate efficient polyfunctional neutralizing activities against different tier 1 and tier 2 HIV-1 strains in accordance with previous data obtained after W614A-3S immunization in mice (Petitdemange et al., 2013), and display Fc-mediated inhibitory functions.

In the absence of fresh memory B cells for these patients to perform a complete characterization of W614A-3S NAbs, we have analyzed their heavy chain complementary-determining region 3 (CDR-H3) motifs to provide insights in accordance with their functional capacities. As previously reported, many of the HIV-1 NAbs have long CDR-H3 sequences, especially those against the glycan-related V3 or the gp120/gp41 bridging regions, and the gp41-MPER motif. In general long CDR-H3 are found in < 0.2% of antibody heavy chains (Yu and Guan, 2014). MS/MS analysis revealed between 3 and 9 fully sequences CDR-H3s for each antibody. Given the complexity and sequence homology of a polyclonal response, it is important to note that from W614A-3S and WT-3S polyclonal mixtures, we cannot exclude that only the most representative deduced amino acid CDR-H3 sequences were obtained. Table S3 shows that purified W614A-3S NAbs from 3 LTNP patients (#02.002, #04.034 and #09.015) have long CDR-H3 sequences (ranged between 22 and 29 residues), which is consistent with the length of control monoclonal NAbs (2F5, 10-1074, 10E8, and PGT-121). In contrast, the length of CDR-H3 sequences of the purified non-neutralizing WT-3S Abs from two patients (#02.011 and #04.061) was inferior to 15 residues. It seems that any dissimilarity in the amino acid sequence account in the neutralizing capacities of the tested Nabs from the 3 LTNP patients (#02.002, #04.034 and #09.015), as described (Xu and Davis, 2000).

### 3.2 W614A-3S Abs Display Neutralizing Activity That is Inversely Associated With Viral Load

**Fig. 2:**
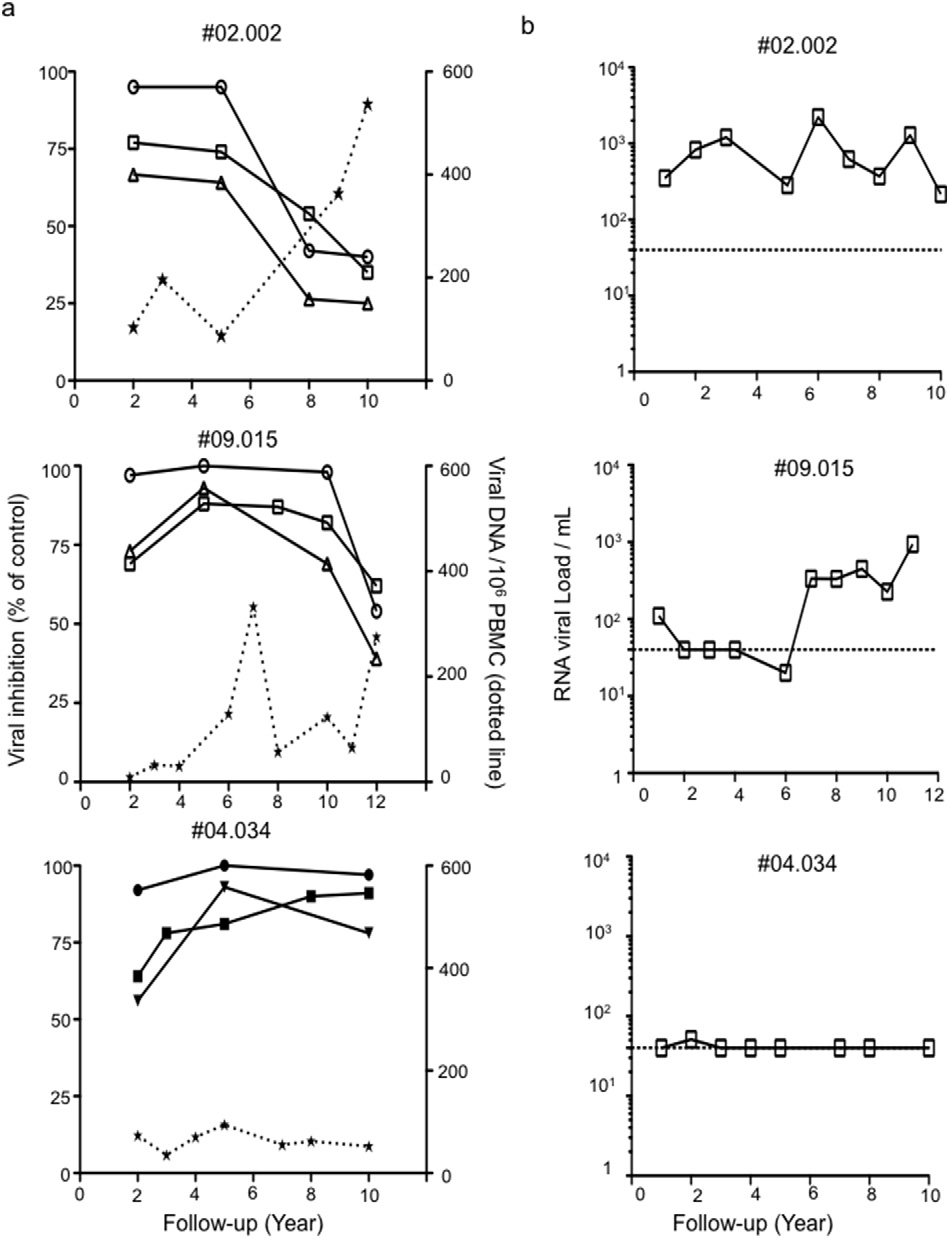
Individual longitudinal neutralizing activity in LTNP patients (#02.002, #09.015 and #04.034) during the follow up. (A) Association between neutralizing activity and viral DNA (star and dotted line) in patients, who produced W614A-3S Abs. Neutralizing activities were assessed with purified Abs, using the TZM-bl assay with SF162 (tier 1, circle), YU-2 (tier 2, square) and QH092 (tier 2, triangle) clade B viruses. Data are expressed as the percentage of decreased RLU values at 1 μg/ml W614A-3S Abs, compared with the RLU detected in infected cells without Abs (control). Similar data were obtained with other HIV-1 strains (NL4.3, NDK, and JR-CSF) (data not shown). (B) Kinetics of RNA viral load evolution.

A central question surrounding the relationship between NAb and clinical outcome remains to be determined. To assess this in the context of in LTNP patients, we looked at the impact of W614A-3S NAb on the viral load. Fig. 2A shows that the persistence of high W614A-3S NAb level in the “controller” (#04.034) is associated with strong control of the viral DNA, whereas the decreased neutralizing activity during the follow up, was observed in the two “viremic” patients (#02.002 and #09.015) with uncontrolled viral DNA. Similar profiles were obtained for RNA viral load (Fig. 2B).

**Fig. 3:**
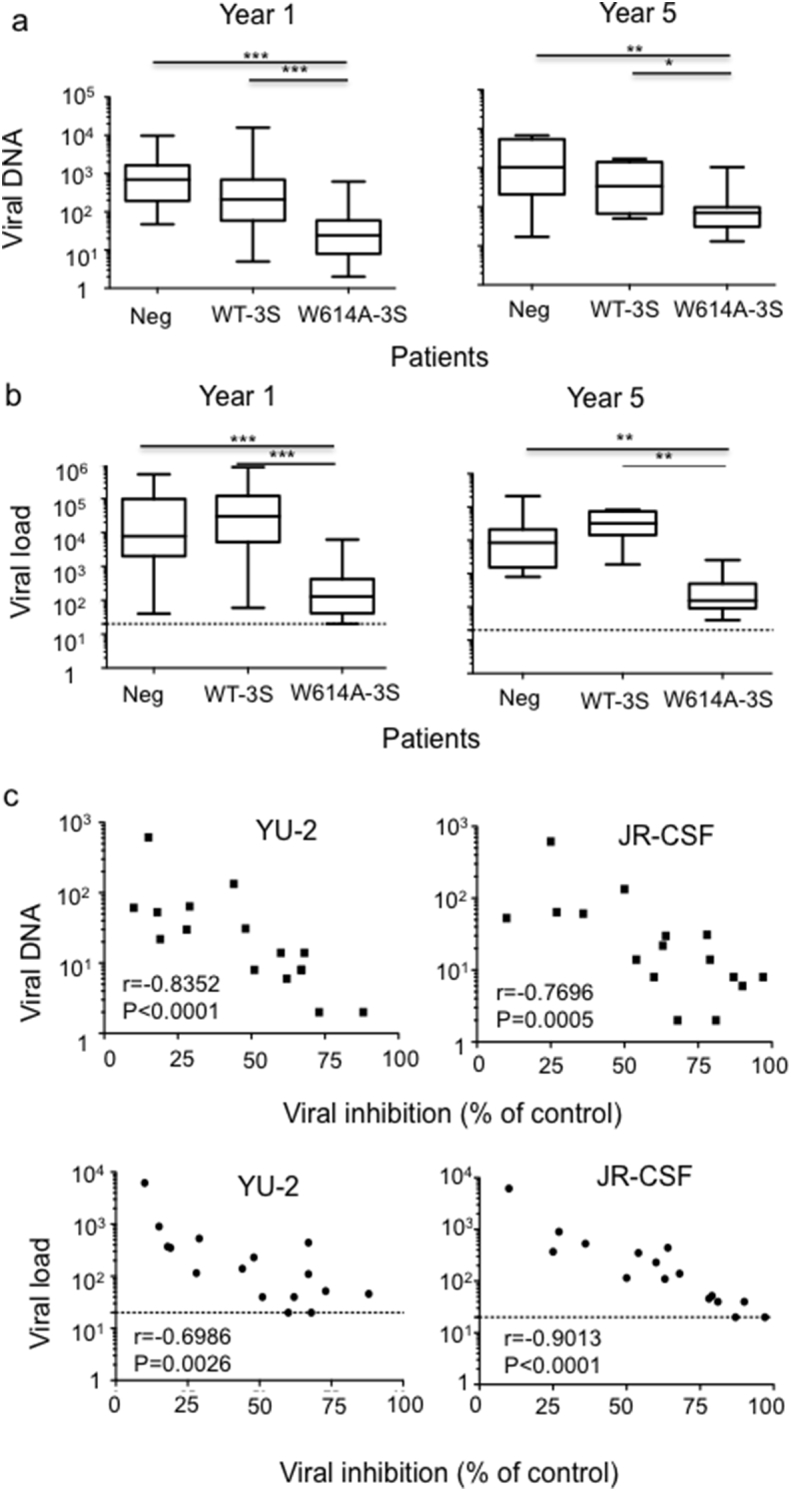
Robust decrease in RNA viral load and viral DNA in LTNP patients producing W614A-3S NAbs. (A) Viral DNA in LTNP patients subdivided into three groups according to their capacity to produce W614A^+^-3S NAbs, non-neutralizing WT-3S^+^ Ab only, or neither subtype (Neg). Data are done at years 1 and 5 after cohort entry. (B) RNA viral load in the different groups of LTNP patients at years 1 and 5 after cohort entry. (C) Correlations between RNA viral load, viral DNA and neutralizing activity of purified W614A-3S NAbs from the 16 ALT patients against JR-CSF and YU-2 HIV-1 strains at year 1 after cohort entry. Neutralizing activity was measured as the percentage of viral inhibition compared with the control. Dotted lines indicate the limit of detection of viral load.

In the entire cohort of LTNP patients, we next search for correlations between the presence of W614A-3S NAbs and viral features. As shown in Fig. 3A, viral DNA was significantly lower in W614A-3S^+^ patients than in control group during the 5-year follow-up (*p* < 0.001). Concomitantly, the viral load was at least 2-log lower in patients with W614A-3S Abs than in the other groups (*p* < 0.001; Fig. 3B). At cohort enrollment, neutralizing activity of purified W614A-3S Abs was significantly and negatively correlated both with RNA viral load (YU-2: *r* = − 0.6986; *p* = 0.0026), and viral DNA (YU2: *r* = − 0.8352; *p* < 0.0001) (Fig. 3C). Similar data were obtained with JR-CSF, another tier-2 HIV-1 strain (Fig. 3C). In contrast, no statistical correlation was measured with purified WT-3S Abs (data not shown), but also with Abs against the T20 peptide, which contains the 2F5 neutralizing core epitope (Fig. S3) (Vieillard et al., 2006). These results show that patients with W614-3S NAbs can be distinguished from the other LTNPs at enrolment by their lower viral levels.

### 3.3 W614A-3S NAbs Correlate with High CD4^+^ T-cell Responses

**Fig. 4:**
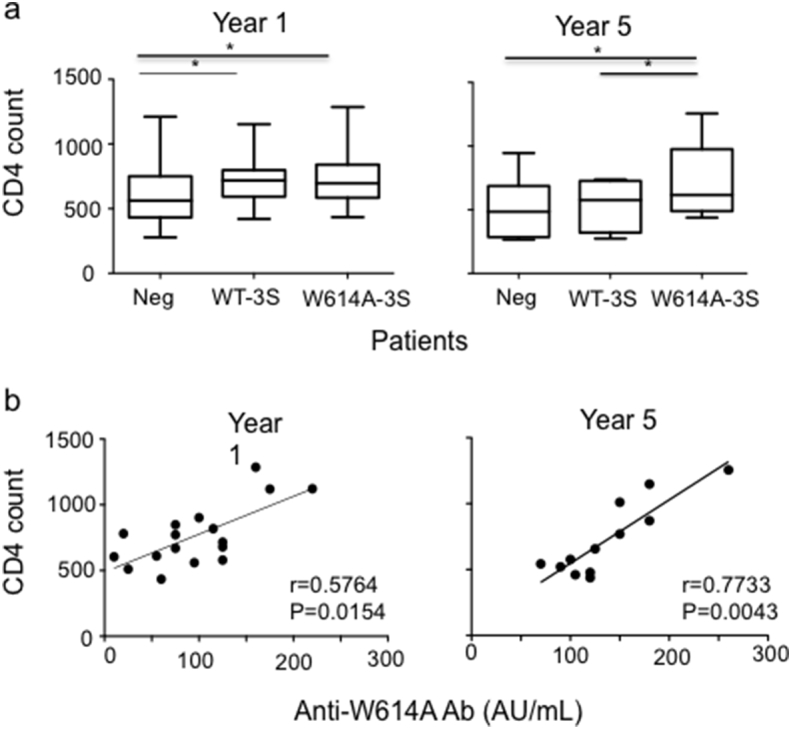
W614A-3S NAbs are associated with high CD4^+^ T-cell counts and continued ALT controller status. (A) CD4^+^ T-cell counts in LTNP patients subdivided into three groups according to their capacity to produce W614A^+^-3S NAbs, non-neutralizing WT-3S^+^ Ab only, or neither subtype (Neg). Data are done at years 1 and 5 after cohort entry. (B) The positive correlation between CD4^+^ T-cell counts and W614A-3S Abs titer at years 1 and 5 after cohort entry.

We then examined whether W614A-3S NAbs affect the T-cell compartment. Fig. 4A shows that the absolute CD4^+^ T-cell count was significantly higher at enrollment in patients with WT- and/or W614A-3S Abs than in patients from the Neg group. This suggests that anti-3S (WT and W614A) Abs could play a positive effect on CD4^+^ T cells, as previously observed in SHIV-infected macaques immunized with 3S peptide (Vieillard et al., 2008, Vieillard et al., 2012). After 5 years, the only patients whose CD4^+^ T-cell counts increased were W614A-3S^+^ (Fig. 4A). The data for the percentage of CD4^+^ T cells and CD4/CD8 T-cell ratios were similar (Fig. S4), whereas no difference were observed in patients producing anti-T20 Abs (Fig. S3). Notably, the level of W614A-3S NAbs was significantly correlated with CD4^+^ T-cell counts at 1 (*r* = 0.5764; *p* = 0.0154), and even more at 5 years (*r* = 0.7733; *p* = 0.0043) after inclusion (Fig. 4B). Moreover, as shown in Table 1, markers of activation (HLA-DR and CD38) and differentiation (CD45Ra/Ro) on CD4^+^ T cells were low in W614A-3S^+^ patients, although not significantly different from the other two groups. By contrast, CD8^+^ T cells from W614A-3S^+^ patients expressed significantly lower levels of HLA-DR^+^ (*p* = 0.007) and CD38^+^ (*p* = 0.03) activation markers than cells from the other two groups (Table 1).

**Fig. 5:**
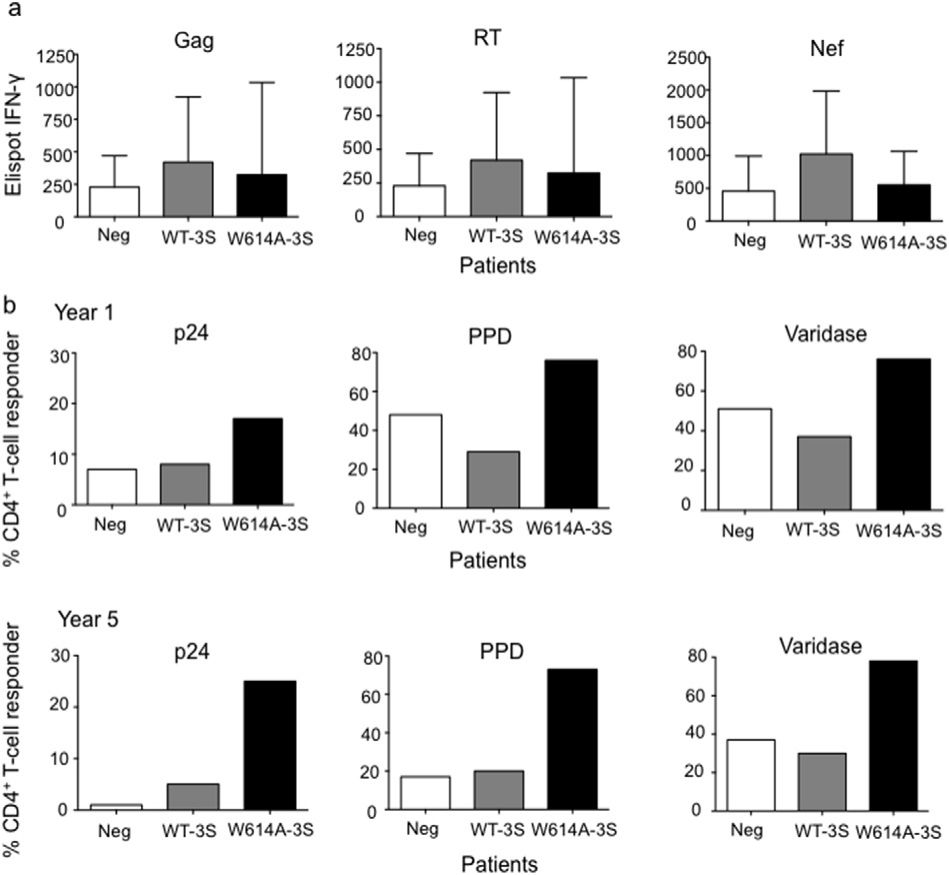
CD4^+^ and CD8^+^ T-cell responses in patients producing neutralizing W614A-3S Abs. (A) CD8^+^ T-cell responses to HIV 15-mer peptides covering Gag, RT, and Nef in ELISpot interferon-γ assays in counts in LTNP patients subdivided into three groups according to their capacity to produce W614A^+^-3S NAbs, non-neutralizing WT-3S^+^ Ab only, or neither subtype (Neg). Data are done at year 1 after cohort entry. Individual sums of positive responses to HIV peptide pools are shown. Results are expressed as spot-forming cells (SFCs)/10^6^ PBMCs. The positive threshold was 50 SFCs/10^6^ PBMCs. (B) CD4^+^ T-cell responses to HIV p24 protein and recall antigens (tuberculin (PPD) and varidase) in a proliferation assay after ^3^H–thymidine incorporation in the different patient groups at years 1 and 5 after cohort entry. A positive response was defined by data > 3000 cpm and a stimulation index > 3. Results are expressed as a percentage of CD4^+^ T-cell responders.

Finally, we evaluated CD4^+^ and CD8^+^ T-cell responses in W614A-3S^+^ patients. The frequency of Interferon (IFN)-γ-producing HIV-specific CD8^+^ T cells was similar in all groups of patients (Fig. 5A). By contrast, the frequency of CD4^+^ T-cell responders to HIV-p24 and different recall antigens tested, was higher in the group of W614A-3S^+^ patients than in the other groups over time (Fig. 5B). This suggests that recall and HIV-specific CD4^+^ T cell responses were specifically preserved in patients who produced W614A-3S^+^ Abs.

## 4 Discussion

Here, we report that NAbs against the highly conserved 3S gp41 Env motif are more prevalent in LTNP HIV^+^ patients than in HIV progressor patients (23.5% *vs* 4.7%) (Gallo et al., 2003), indicating that this motif is likely associated with a lack of disease progression. Furthermore, W614-3S NAbs were detected predominantly in patients with low levels of viral load and viral DNA and high CD4^+^ T-cell counts and preserved T-cell function. The absence of effect mediated by anti-T20 Abs suggests that function associated with anti-W614A-3S NAb is certainly not a general property of NAbs, and support the hypothesis that W614A-3S NAbs provide immune benefits and may be associated with LTNP status. We can however not exclude that W614A-3S Abs develop preferentially under conditions of non-progression, such as high CD4^+^ T cells and low viral load.

NAbs occurring after initial infection with HIV-1 are generally not associated with any clinical or biological benefits. Doria-Rose et al. (2010) suggested that the limited breadth of neutralization observed in whole-serum samples from LTNP patients maybe attributable to low levels of antigenic stimulation due to their undetectable viral loads. Indeed, several studies have demonstrated that the development of NAbs is significantly associated with duration of infection and high viral load and that NAbs do not protect against HIV-1 disease progression, whereas Freund et al. (2017) have recently shown the coexistence of potent NAbs and antibody-sensitive viruses in a viremic controller. These findings were consistent with a model in which antigenic stimulation drives the breadth of the NAb response (Euler et al., 2010, Piantadosi et al., 2009). Such a model, however, is not supported by our data. Notably, previous studies examined neutralizing activities in whole-serum samples and not in samples specifically prepared from highly conserved motifs of Env. Interestingly, the neutralizing activity of W614A-3S-specific Abs observed in the TZM-bl standard assay was greatly potentiated by targets expressing FcγRI, as previously observed for MPER-specific mAbs (Perez et al., 2009). The mechanism remains unclear, but our pre-positioning of the 3S motif at the cell surface, where it has been observed on the pre-fusion HIV envelope 3D structure (Fig. S1), may have facilitated its binding to FcγRI, thereby conferring a kinetic advantage for virus inhibition by W614A-3S Abs. Generally, this could be observed in Abs with epitopes exposed for at least a short time on an intermediate pre-fusion conformation of gp41 (Wyatt and Sodroski, 1998, Labrijn et al., 2003). FcγRI engagement may also stabilize a favorable orientation of Abs at the sterically constrained virus-cell interface to provide an additional kinetic advantage for a specific intermediate conformation of gp41. This will have to be investigated, however, this hypothesis is strengthened by the high conservation of the W614 position within the 3S motif of the gp41 in all LTNP tested (Table S1) (Potard et al., 2013), suggesting that W614A-3S Abs could recognize a “conformational intermediate epitope”, mimic by the W614A-3S peptide. Thus, the generation of W614A-3S Abs is certainly not the result of mutated W614A-3S virus production, which are non-infectious (Petitdemange et al., 2013), but could be associated with an increased Ab binding and/or neutralizing breadth.

Although rare on CD4^+^ lymphocytes, FcγRs are highly expressed by macrophages and dendritic cells, which are involved in the sexual transmission and early establishment of long-lived viral reservoirs^25^. We observed greater neutralizing activity in macrophages (MDMs) than in TZM-bl cells, as previously reported for HIV and SIV infections with MPER mAbs (Perez et al., 2013, Lederle et al., 2014, Su and Moog, 2014, Zhuge et al., 1997, Ruppach et al., 2000). Remarkably, the polyclonal neutralizing efficiency of W614A-3S NAbs against the BaL HIV strain in MDMs was highly potent, with levels similar to or higher than those observed with neutralizing mAbs. These polyfunctional capacities may be extremely useful for a prophylactic vaccine.

To date, most studies have analyzed NAbs cross-sectionally, and only a few have conducted long-term follow-up of neutralizing responses according to HIV-1 disease outcome (Cecilia et al., 1999, Chaillon et al., 2012). Here, we observed that the neutralizing activity of purified W614A-3S Abs increased in all LTNP patients at 5 years post-enrollment. As the purified W614A-3S Abs corresponded to a mixture of mAbs, as suggested by the variation in term of CDR-H3 motifs, it is tempting to speculate that affinity maturation leading to the selection for the most efficient NAbs occurred with time. Notably, W614A-3S Ab-mediated neutralizing activity was both inversely correlated with viral load and viral DNA, in accordance with the enhanced clearance of infected cells after passive transfer of NAbs in SHIV-infected macaques (Lu et al., 2016, Liu et al., 2016). Notably, W614A-3S Ab neutralizing activity decreased in LTNP patients with persistent viral loads, perhaps due to modifications of Ab/epitope recognition following viral escape mechanisms over time, as described (van Gils et al., 2010). Our results suggest that the durability of certain NAbs may be a major clue to the mechanisms of long-term non-progression. To date however, the mechanism to explain the presence of W614A-3S NAb remains unclear, we can however hypothesized that W614A-3S Abs recognized an “intermediate conformational epitope” within the highly conserved 3S motif of the gp41, which is mimic by the W614A-3S peptide. In accordance with the substantial conformational changes in gp41 used facilitate viral entry (Wyatt and Sodroski, 1998), we can supposed that this “intermediate conformational epitope” should be exposed for at least a short time on an intermediate pre-fusion conformation of gp41. A complete characterization of W614A-3S NAb could be very informative.

The decrease in Ab levels is undoubtedly caused by general deterioration of the immune response, which is well documented, not only in terms of decreased CD4^+^ T-cell counts but also in proliferative responses (Imami et al., 2013, Lewis et al., 2014). Here, we sought to quantify and characterize peripheral CD4^+^ and CD8^+^ T-cell populations that may be correlated with the neutralizing activity of W614A-3S Abs. As we previously reported, anti-3S Abs are clearly associated with higher CD4^+^ T-cell counts and decreased cell activation (Vieillard et al., 2012, Petitdemange et al., 2013). Studies using animal models have established the importance of virus-specific CD4^+^ T cells in the induction and maintenance of effective host immune responses (Migueles and Connors, 2015). In HIV-1 infection, several features of HIV-specific CD4^+^ T cells, including proliferative capacity, surface phenotype, and secretion of multiple cytokines, are impaired in individuals with high plasma concentrations of HIV-1 RNA. However, cART can improve these features to resemble those observed in LTNP patients (Emu et al., 2005). Here, we demonstrated that CD4^+^ T cells from LTNP patients producing W614A-3S NAbs improved their functional capacities such that they could specifically recognize antigens from both HIV itself and recall antigens. Simultaneously, viremia decreased significantly, as previously observed in patients with long-term AIDS-free infection (Carotenuto et al., 1998). CD4^+^ T-cell responses to HIV antigens appear to be selectively impaired during high-level viremia and may be restored when HIV replication is controlled using therapy (McNeil et al., 2001, Lange et al., 2002). Conceivably, W614A-3S Abs participate in the protection of LTNP patients by continuous virological control, resulting in maintenance of CD4^+^ T-cell count and function.

Evidence suggests that the frequency of broad CD8^+^ T-cell responses is high in LTNP patients (Imami et al., 2013), as observed in the W614A-3S^+^ and other patients in this study. However, W614A-3S^+^ HIV-infected individuals had lower numbers of CD8^+^ T cells, and these cells were less activated. Discordant expression of these activation markers has also been documented on HIV-specific cells of LTNP patients (Sáez-Cirión et al., 2007, Lécuroux et al., 2014). Notably, we observed that CD8^+^ T-cell activation decreased over time in W614A-3S^+^ patients due to their low viral load, as described (Imami et al., 2013, Wherry et al., 2003).

This study provides strong arguments that W614A-3S NAbs contribute to LTNP status, although, *in vivo* immunization or passive-transfer experiments with purified W614A-3S Ab in macaques will have to be investigated to determine their “protective” value. In addition to their putative role in a prophylactic vaccine strategy, we hypothesized that W614A-3S Ab, like other Nabs, could be also investigated as possible therapeutic agents in accordance with recent studies demonstrate that monoclonal Abs significantly reduced viremia in chronically infected macaques (Stephenson and Barouch, 2016), suggesting that such therapies might be effective in humans. Thus, a combination of conventional multi-hits antiretroviral therapy with NAbs therapy might be successful and could generate a strategy that may lead to an HIV cure. Future clinical approaches for inducing W614A-3S NAbs should be developed alone or in combination as steps toward both a functional cure by immunotherapy and prophylactic vaccines.

## Conflicts of Interest

The authors declare no conflicts of interest.

## Author Contributions

O.L. and B.S. performed experiments, interpreted and analyzed data. V.P. performed statistical analysis. A.S., B.A., P.D. interpreted and analyzed data. C.M. designed assays, interpreted and analyzed data and edited the manuscript. V.V. designed assays, interpreted and analyzed data, wrote and edited the manuscript.

## Appendix A Supplementary data

Supplementary material Image 1
