# Supplementary material for: Neutralizing Antibodies Against a Specific Human Immunodeficiency Virus gp41 Epitope are Associated With Long-term Non-progressor Status
Source: eBioMedicine. 2017 Jul 11;22:122–32. doi: 10.1016/j.ebiom.2017.07.007 (PMC5552210; doi:10.1016/j.ebiom.2017.07.007)
Supplement: Supplementary file 1 — Supplementary material [file mmc1.doc]

**Lucar et al.**

**Supplemental Materials**

**Table S1. Sequencing of the 3S motif in Neg-, WT^+^- and W614A^+^-3S groups of LTNP samples**

LTNP patients are subdivided into three groups according to their capacity to produce neutralizing W614A-3S Ab (W614A^+^-3S), non-neutralizing WT-3S Abs (WT^+^-3S), or neither subtype (Neg-3S). nd: not done; The position 614 is noted in red

**Table S2. Neutralizing activity of potent human monoclonal antibodies**

| Samples | IC_80_ (μg/ml) | | | | |
| --- | --- | --- | --- | --- | --- |
|  | TZM-bl assay | | | | MDM assay |
|  | SF162 | QH092 | pCH058 | pCH077 | BaL |
| VRC01 | 1 | 5 | nd | nd | 0.005 |
| VRC07 | 0.25 | 3 | nd | nd | 0.01 |
| 3BNC117 | 0.6 | 2 | 0.3 | 0.5 | 0.005 |
| PG121 | 0.02 | 2 | <0.3 | 20 | 0.004 |
| 447-52D | 4 | >20 | nd | nd | 0.04 |
| 2F5 | 4 | 5 | nd | nd | 0.005 |
| 2G12 | 4 | 5 | nd | nd | 0.2 |
| IgG1b12 | 0.4 | 5 | nd | nd | 1 |
| 10-1074 | nd | nd | 0.3 | 20 | nd |
| 10-E8 | nd | nd | 3 | 5 | nd |

A orange-color scale indicates ranges of values for maximal concentration needed to neutralize 80% of virus infection (IC_80_). nd: not done

**Table S3. Deduced amino acid CDR-H3 sequences of purified W614A-3S Ab from LTNP patients**

Candidats CDR-H3 sequences Size^§^

___________________________________________________________

**Monoclonal NAbs***

10-1074 CATARRGQ--RIYGVVSFGE---FFYYYSMDVW 28

PGT121 CARTLHGR--RIYGIVAFNE---WFTYFYMDVW 28

2F5 CAHRRGPT--TLFGVPIAR-----GPVNAMDVW 26

10E8 CARTGKYYDF-WSGYPP--------GEEYFQDW 24

**WT-3S Abs**

02.011a C-R-G--YNDDF-T-------------YI-DVW 14

02.011b --RSG-Y--DDF----------------VDMVW 12

02.011c C-RS--Y--NDF----------------ID-VW 11

02.011d C-RT--YY-N-V----------------I--VW 10

02.011e --R-TGDYDNDF----------------V-MDW 13

02.011f C-R-GGD-TDDF----------------ID--W 12

04.061-a -TRSY--IVD-F-----------------FDVW 12

04.061-b --RSI--YGD-----------------IV-DVW 11

04.061-c CTR----SDD-Y---------------IVD-VW 12

**W614A-3S NAb**

02.002-a -AHTTQSRY--SFGSNEGDLMTTLESQY--QDW 27

02.002-b CAHTTQRY---SFGFIIGD-MTTLESQI--QDW 27

02.002-c CAHTTQRY---SFGFNEGDLMTTLESGYPIQDW 29

04.034-a CAHTTQRSSY------NEDIIMTLESGY--QDW 25

04.034-b -AHTTQRY---SFGMTT------LE--S--DDW 19

04.034-c --HTTQRS---SFGIIVDL-MTTLESGY--QDW 24

04.034-d CAHT-QRY---SF----GD-MTTLESPY--QDW 22

09.015-a CA-TTQRY---SF----GD-MTTLESGY--QDW 22

09.015-b -AHTRTSF--GNE-----DLMMTL-IGY--QDW 22

09.015-c CAHTQRSF--GYYGI-IVDLMT-LESPY--Q-W 25

09.015-d -AHTQRS----YYG--IIDLMTTLESPY--DDW 22

09.015-e C-HT-MLTT----------QQGIIESGYPEQDW 21

09.015-f CAHTTQRG---YYGF--GDIIMT-LEGYPIQDW 26

09.015-g --HTQRGY---F-GFIIDGDMTTLESGYPIQDW 27

09.015-h CAHTTQRGYL-LYG-----FMTTLES-Y--DVW 24

09.015-i -AATTQSSFFLLYGFIIGD-MT-LESGY--QDW 28

___________________________________________________________

* Data from the broadly neutralizing antibodies electronic resource (bNaber; [http://www.bnaber.org](http://www.bnaber.org/)).

§ Size is expressed in number of amino-acid residues; Capital letters represent amino-acids and dashes represent gaps in the alignment. Alignment was assessed with Clustal Omega.

**
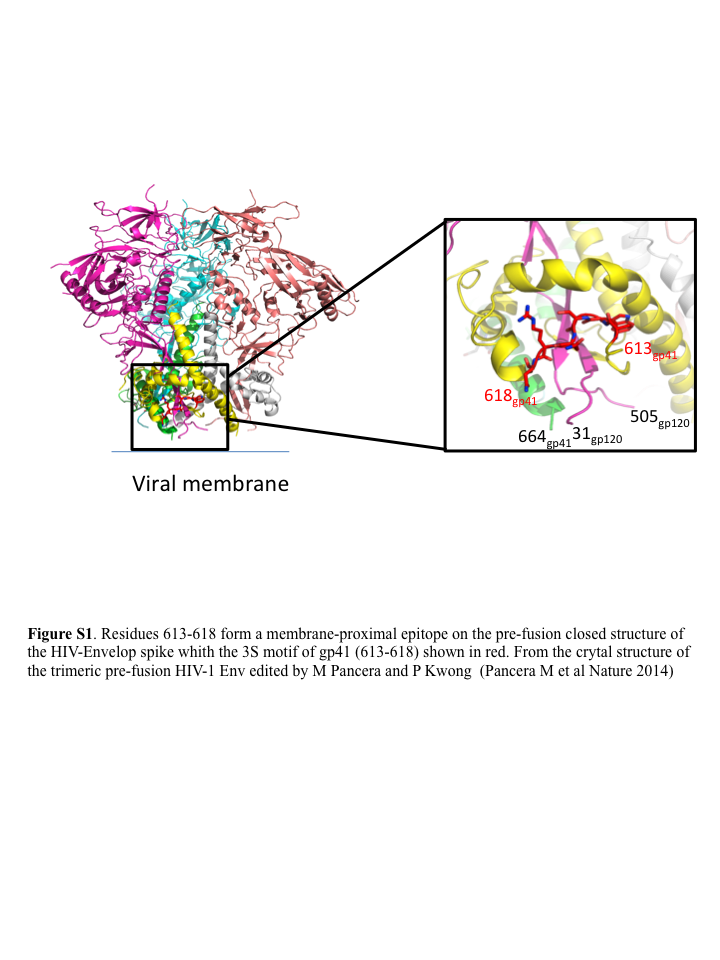
**

**Figure S1.** Residues 613-618 of the 3S motif form a membrane-proximal epitope on the pre-fusion closed structure of the HIV-Envelop spike. The 3S motif of gp41 (613-618) shown in red (right panel). Adapted from the crystal structure of the trimeric pre-fusion HIV-1 Env (Pancera et al., 2014).

**
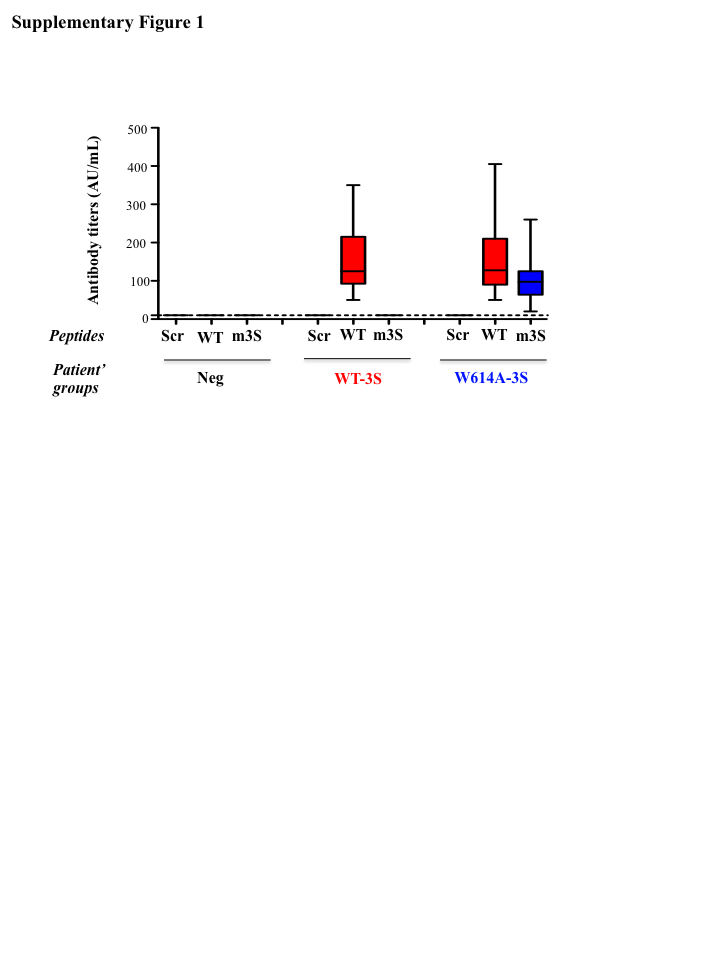
**

**Figure S2.** Subdivision of the 68 LTNP patients into three groups according to their capacity to produce neutralizing W614A-3S Abs, non-neutralizing WT-3S Abs only, or neither subtype (Neg). Plasma samples were titrated by ELISA with purified WT peptide (NH2-pwnasSWSNKSssleqiw-COOH) to quantify WT-3S Abs, with the m3S peptide to quantify W614A-3S Abs (NH2-pwnasSASNKSssleqiw-COOH), or a scramble peptide (Scr; NH2-pnsakwlwssiqsnswes-COOH) as control.

**
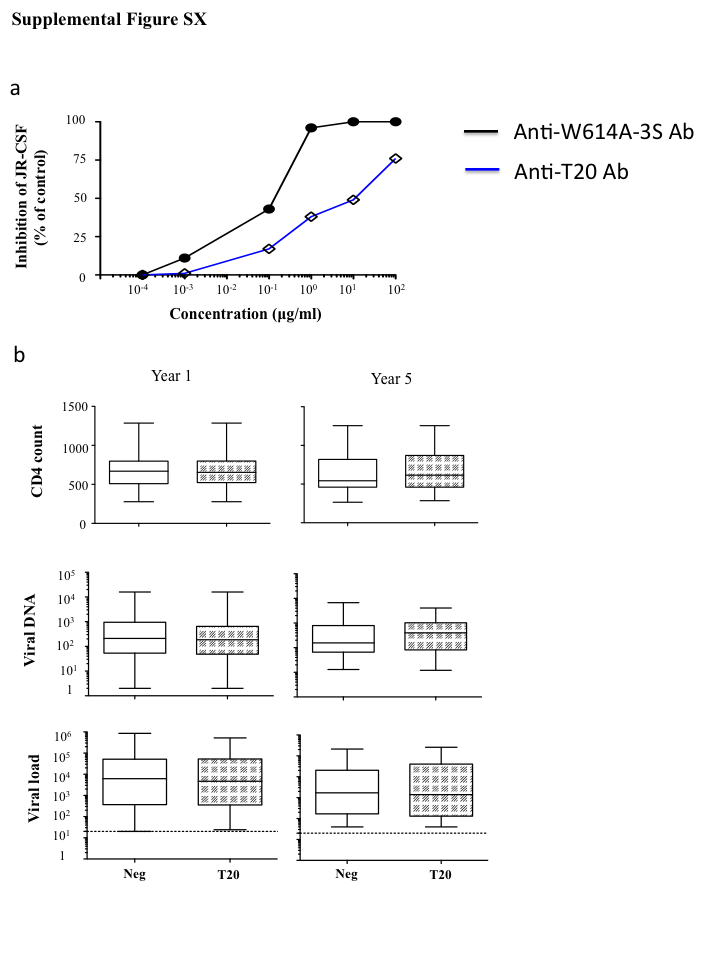
**

**Figure S3.** Neutralizing activity in the Absence of immunological and virological modulations in LTNP patients producing anti-T20 Abs (Vieillard et al., 2006). (A) Dose-response curves of neutralizing activity . Inhibition of infection by JR-CSF HIV-1 strain, at different concentrations with purified W614A-3S Abs (closed symbols, black curves) or T20 Abs (open symbols, blue curves) from LTNP patients. (B) CD4 count, viral DNA and RNA viral load in LTNP patients producing (T20, dashed whiskers) or not (Neg, open whiskers) anti-T20 Abs. Data are done at years 1 and 5 after cohort entry.

**
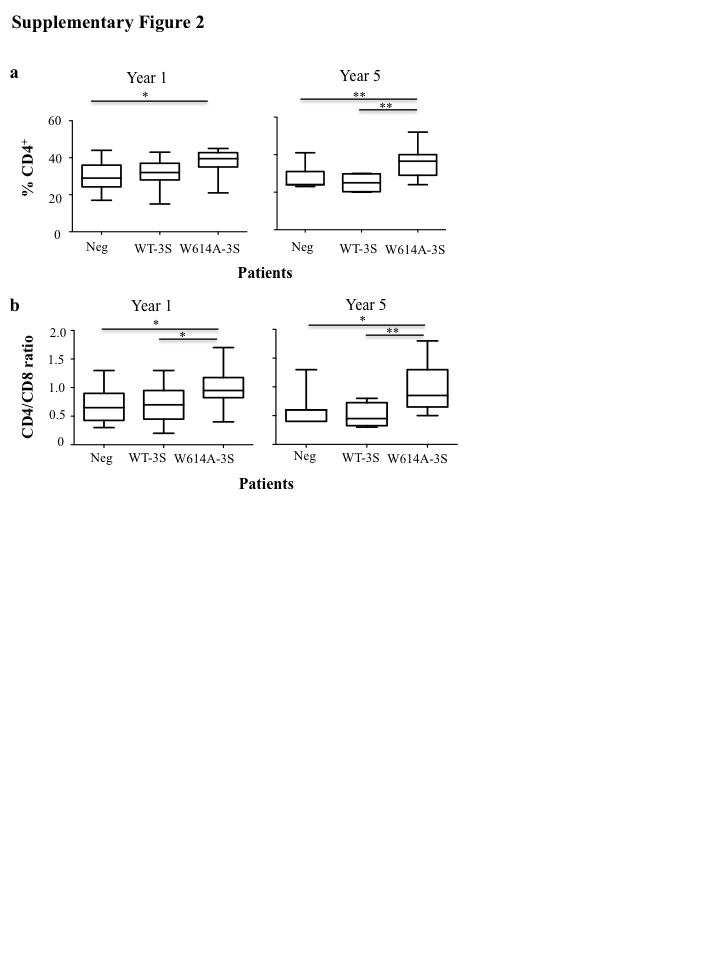
**

**Figure S4.** Frequency of CD4^+^ T cells (A), and CD4/CD8 ratio (B) at years 1 and 5 after cohort entry, in three groups of patients according to their capacity to produce neutralizing W614A-3S Ab, non-neutralizing WT-3S Abs, or neither subtype (Neg).
